# IDEAL-IQ combined with intravoxel incoherent motion diffusion-weighted imaging for quantitative diagnosis of osteoporosis

**DOI:** 10.1186/s12880-024-01326-0

**Published:** 2024-06-20

**Authors:** Zhe Yang, Chenglong Liu, Zhaojuan Shi, Jian Qin

**Affiliations:** Department of Radiology, The Second Affiliated Hospital of Shandong First Medical University, Tai’an, Shandong 271000 China

**Keywords:** IDEAL-IQ, IVIM-DWI, Osteoporosis, Bone mineral density

## Abstract

**Background:**

Osteoporosis (OP) is a common chronic metabolic bone disease characterized by decreased bone mineral content and microstructural damage, leading to increased fracture risk. Traditional methods for measuring bone density have limitations in accurately distinguishing vertebral bodies and are influenced by vertebral degeneration and surrounding tissues. Therefore, novel methods are needed to quantitatively assess changes in bone density and improve the accurate diagnosis of OP.

**Methods:**

This study aimed to explore the applicative value of the iterative decomposition of water and fat with echo asymmetry and least-squares estimation-iron (IDEAL-IQ) sequence combined with intravoxel incoherent motion diffusion-weighted imaging (IVIM-DWI) for the diagnosis of osteoporosis. Data from 135 patients undergoing dual-energy X-ray absorptiometry (DXA), IDEAL-IQ, and IVIM-DWI were prospectively collected and analyzed. Various parameters obtained from IVIM-DWI and IDEAL-IQ sequences were compared, and their diagnostic efficacy was evaluated.

**Results:**

Statistically significant differences were observed among the three groups for FF, R2*, f, D, DDC values, and BMD values. FF and f values exhibited negative correlations with BMD values, with *r*=-0.313 and − 0.274, respectively, while R2*, D, and DDC values showed positive correlations with BMD values, with *r* = 0.327, 0.532, and 0.390, respectively. Among these parameters, D demonstrated the highest diagnostic efficacy for osteoporosis (AUC = 0.826), followed by FF (AUC = 0.713). D* exhibited the lowest diagnostic performance for distinguishing the osteoporosis group from the other two groups. Only D showed a significant difference between genders. The AUCs for IDEAL-IQ, IVIM-DWI, and their combination were 0.74, 0.89, and 0.90, respectively.

**Conclusions:**

IDEAL-IQ combined with IVIM-DWI provides valuable information for the diagnosis of osteoporosis and offers evidence for clinical decisions. The superior diagnostic performance of IVIM-DWI, particularly the D value, suggests its potential as a more sensitive and accurate method for diagnosing osteoporosis compared to IDEAL-IQ. These findings underscore the importance of integrating advanced imaging techniques into clinical practice for improved osteoporosis management and highlight the need for further research to explore the full clinical implications of these imaging modalities.

## Key point

IVIM-DWI’s Diagnostic Value: IVIM-DWI, especially its parameter D, showed high efficacy for osteoporosis diagnosis. Its ability to separate diffusion from microcirculatory perfusion offers advantages over traditional methods like DXA, potentially enabling earlier and more precise detection of osteoporosis and guiding timely interventions.

Superiority of IVIM-DWI: IVIM-DWI outperformed IDEAL-IQ in osteoporosis diagnosis. Its capability to provide comprehensive information about bone density and microstructural changes makes it a valuable addition to imaging protocols, aiding clinicians in making more informed decisions and personalized treatment plans.

Demographic Influences: Significant differences in imaging parameters were observed between genders and age groups. Understanding these demographic influences can enhance the interpretation of imaging data, guiding tailored management strategies for specific patient populations and potentially shedding light on the underlying mechanisms of osteoporosis.

## Background

Osteoporosis (OP) is a chronic [1] metabolic bone disease characterized by decreased bone mineral content and partial damage of the bone microstructure [2], resulting in disrupted bone formation and absorption [3], and increased susceptibility to fractures [4, 5]. The World Health Organization recommends bone mineral density (BMD) measurement at the lumbar spine or femoral neck using dual-energy X-ray absorptiometry (DXA) as the gold standard for diagnosing OP. However, the clinical application of DXA is limited by the fact that it could not clearly distinguish between lumbar vertebrae and adjacent anatomical structures, such as soft tissues and organs, due to inadequate contrast resolution. As a common imaging method, conventional MRI can be used for the diagnosis of the vertebral diseases, with characteristics of high resolution, no radiation and multiparameter. However, solely relying on visual evaluation of signal changes in traditional MRI may not provide sufficient accuracy in confirming osteoporosis, highlighting the need for new quantitative methods to evaluate changes in BMD accurately. This emphasizes the importance of exploring advanced imaging techniques such as IDEAL-IQ and IVIM-DWI, which offer quantitative assessments and may address the urgency for more precise diagnostic approaches in osteoporosis diagnosis. The Iterative Decomposition of Water and Fat with Echo Asymmetry and Least-Squares Estimation-Iron (IDEAL-IQ) sequence represents a state-of-the-art, three-dimensional imaging [6] technique [7] developed based on the Dixon [8] method. By employing water-lipid separation and recombination, IDEAL-IQ generates six sets of images [9], including fat fraction (FF) images and R2* images [10]. This advanced approach effectively mitigates the influence of main magnetic field uniformity and addresses issues arising from factors such as T2 relaxation effects [11, 12]. Consequently, it enables precise evaluation of lumbar vertebral fat [13, 14] and iron content, providing accurate insights into changes in bone mass.

Diffusion-weighted imaging (DWI), initially proposed by Le Bihan [15–18] and his colleagues, serves as a powerful tool to accurately assess the pathological state by examining the degree of diffusion restriction of water molecules [19] within tissues. However, it is important to note that the attenuation of DWI signal also reflects the status of capillary perfusion [20]. A recent advancement in this field is the introduction of intravoxel incoherent motion diffusion-weighted imaging (IVIM-DWI), which enables the differentiation between the diffusion of water molecules and that influenced by microcirculatory perfusion [21–24]. Through biexponential analysis of IVIM-DWI signals, it becomes could obtain the true diffusion coefficient (D), perfusion-related diffusion coefficient (D*), distributed diffusion coefficient (DDC) and perfusion fraction (f) [25]. These parameters offer a more precise reflection of pathological changes [26] within bone tissues. While this imaging sequence has found extensive application in diagnosing various diseases including brain tumors [27], liver fibrosis [28] and breast tumors [29], its utilization in studying vertebrae remains relatively limited [30, 31].

Although dual-energy X-ray absorptiometry (DXA) is recognized by the World Health Organization as the gold standard for diagnosing osteoporosis (OP), its clinical application is subject to limitations. Specifically, the diagnostic accuracy of DXA may be challenged when faced with extensive degenerative changes or artifacts in the spine. Additionally, when evaluating hip bone density using DXA, there are numerous technical limitations and challenges related to anatomical complexity, which may affect its accurate assessment of OP. Therefore, there is an urgent need to research and develop more precise diagnostic methods to address these limitations of DXA. Furthermore, despite the widespread use of DXA for assessing hip bone density, there are also a series of challenges in this area. The complexity of hip anatomy and the influence of surrounding soft tissues may affect the accurate assessment of hip OP using DXA. Moreover, technical challenges similar to those encountered in the spine may also affect the application of DXA in the hip region. Hence, accurate assessment of hip OP requires research and development of more precise diagnostic methods. These two sequences facilitate a more nuanced exploration of diseases at the micro level by allowing for quantitative detection of bone composition. This advancement significantly advances research on related diseases. However, there has been a scarcity of studies comparing the diagnostic efficacy of various parameters for osteoporosis (OP). Consequently, the relationships between these parameters and bone mineral density (BMD) remain contentious. Furthermore, few studies have attempted to compare the diagnostic value of these parameters for OP between the two sequences. In this quantitative study, we have combined IDEAL-IQ and IVIM-DWI to delve into their respective diagnostic value for OP.

## Materials and methods

### Subjects

A total of 135 patients (54 males and 81 females, mean age 61.73 ± 8.86 years) who underwent DXA, IDEAL-IQ and IVIM-DWI at the Second Affiliated Hospital of Shandong First Medical University from October 2022 to March 2023 were included. All the patients’ height and weight were recorded, and their body mass index (BMI) were calculated.

### Inclusion and exclusion criteria

Inclusion criteria: (1) Those who have undergone DXA and MRI check for lumbar vertebra; (2) No relevant intervention or medication treatment was performed before examinations; (3) Patients can lie supine for a long time; (4) Patients have no MRI contraindications, and no pelvic metal artifacts were found on images; (5) Patients with clear and interpretable images, as assessed by experienced radiologists or imaging specialists.

Exclusion criteria: (1) Presence of fractures of the lower thoracic or lumbar vertebrae; (2) Presence of tumor-like lesions or bone destruction in the lumbar spine; (3) Patients taking medications or medical conditions that may affect bone metabolism, such as glucocorticoid hormone, thiazolidinediones and antiepileptic drugs; (4) Patients with suboptimal image quality. This study was conducted in accordance with the principles outlined in the Declaration of Helsinki and approved by the Institutional Review Board of the Ethics Committee of the Second Affiliated Hospital of Shandong First Medical University. Patient confidentiality and data privacy were strictly maintained throughout the study process.

### DXA

DXA (General Electric, USA) was used for the examination of lumbar spine bone density. The L1-4 vertebral bodies of all subjects were scanned to obtain the T-Score and BMD value. According to the diagnostic criteria proposed by WHO, T-Score ≤ -2.5, -2.5 < T-Score <-1.0 and T-Score ≥ -1.0 were diagnosed as osteoporosis, osteopenia and normal, respectively.

### MRI

The lumbar spine was scanned using a 3.0T MR scanner (General Electric, USA). All subjects were scanned in a supine position, and the scanning sequence and parameters were shown in Table 1. Sagittal FSE T1WI and T2WI sequences were used to evaluate changes in lumbar morphology and signal. To ensure objectivity and accuracy in the evaluation, we employed a double-blind assessment method. During the interpretation of MRI images, radiologists were unaware of the patients’ DXA results. Specifically, prior to the assessment of MRI images, information regarding DXA results was isolated to ensure that the assessment process remained unaffected by DXA results. This double-blind design helped minimize subjective biases and ensured objectivity throughout the evaluation process.

**Table 1:** MRI scanning sequence and parameters

SE	IDEAL-IQ	IVIM-DWI	FSE T1WI	FSE T2WI
Position	Sagittal plane	Axial plane	Sagittal plane	Sagittal plane
TR (ms)	8.6	2800	400	2500
TE (ms)	1.6–5.8	68	8	100
Slice thickness (mm)	3	3	3	3
Layer spacing (mm)	1	1	1	1
B values (s/m^2^)	-	0, 50, 100, 150, 200, 400, 600, and 800	-	-

### Image data processing and analysis

**Fig. 1:**
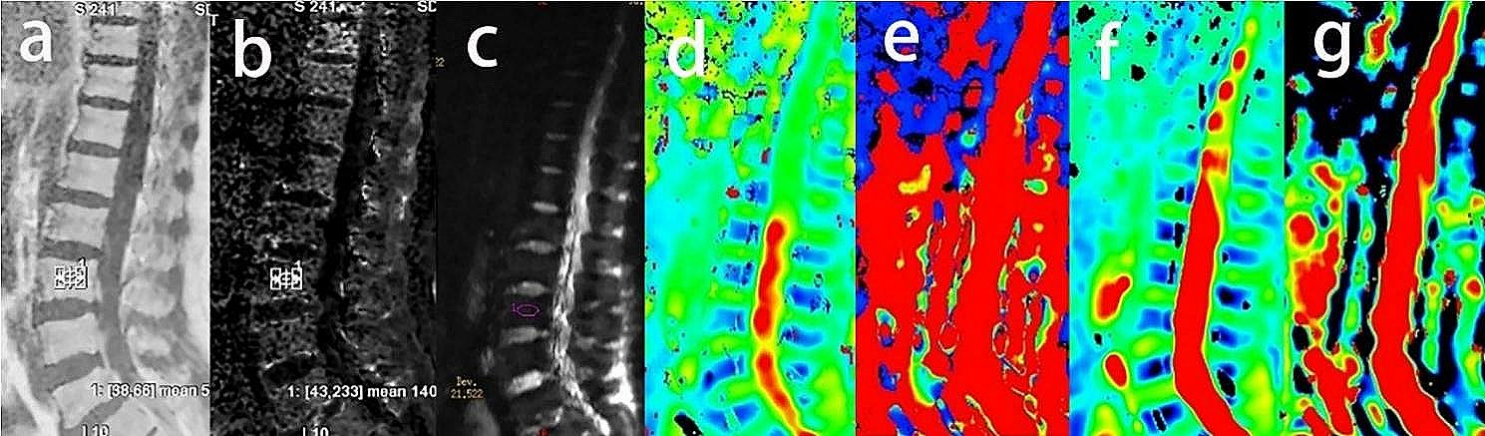
Typical image from a healthy participant without osteopenia or osteoporosis. **a-g** show the FF, R2*, DWI, D, D*, f, and DDC images of the lumbar spine, respectively. The circle on the vertebral body represents the measurement range. The results of **d-g** were shown in the left side of these original images through measuring on image c

The vendor-supplied Functool software from AW4.6 post-processing workstation was utilized for processing, analyzing and calibrating the raw data. A radiologist with over 10 years of experience in MR imaging diagnosis reviewed the routine MR sequence scanning images and excluded cases that did not meet the inclusion criteria. Prior to the formal measurement of parameter values by two postgraduate students, we conducted an intraclass correlation coefficient (ICC) test on the results of twenty vertebrae measured by both students. Regions of interest (ROIs) were placed in the center of the L1-4 lumbar vertebral cancellous bone in the middle slice of sagittal DWI images with b = 0 s/mm², while avoiding the vertebral vein running area and the cortical area. Subsequently, the average values of D, D*, f, and DDC were calculated (Fig. 1c-g). Using the IDEAL-IQ map images, the signal intensities of the same ROIs were calculated to obtain FF and R2*values (Fig. 1a-b).

### Statistical analysis

SPSS26.0 statistical software was utilized for data processing and analysis. Continuous data were presented as mean ± standard deviation and median (interquartile range), with comparisons conducted using either the independent student’s t-test or Mann-Whitney U-tests, as appropriate. Single-factor analysis of variance and the Kruskal-Wallis H-test were employed to assess differences in MRI parameter values, BMI and BMD among the three groups, with subsequent pairwise comparisons conducted using the Least Significant Difference (LSD) t-test and Mann Whitney U-test.

Correlations between BMD, MRI parameters and BMI were examined through Spearman’s correlation analysis. The discriminative performance of various parameters for diagnosing OP was evaluated using receiver-operating characteristic (ROC) curves, with an area under the curve (AUC) ranging between 0.5 and 0.7 indicating low diagnostic capability, 0.7–0.9 indicating moderate capability, and > 0.9 indicating high capability. AUC values from ROC curves were compared using the Delong’s test. A two-sided P-value < 0.05 was considered statistically significant.

## Results

### Patient characteristics

A total of 135 patients (comprising 675 vertebral bodies) were included in the study, consisting of 52 patients diagnosed with osteoporosis (involving 260 vertebral bodies), 45 patients with osteopenia (225 vertebral bodies), and 38 patients categorized as normal (190 vertebral bodies). Of these patients, there were 54 male patients (encompassing 270 vertebral bodies) and 81 female patients (constituting 405 vertebral bodies). The distribution across groups was as follows: 17 males and 21 females in the normal group, 22 males and 23 females in the osteopenia group, and 15 males and 37 females in the osteoporosis groups. The average ages across these groups were 61.53 ± 80.2, 57.47 ± 8.67, and 65.56 ± 7.97, respectively. The average BMI values were 25.72 ± 2.45, 25.44 ± 2.64, 23.87 ± 4.11. T test was used to compare these values among these groups. For ages, there were all statistically different among these groups. For BMI, there were statistically different between normal and osteoporosis groups, osteopenia and osteoporosis groups.

### Consistency analysis

The FF, R2*, D, D*, DDC and f values measured by two students exhibited high consistency, with ICCs of 0.82, 0.80, 0.82, 0.78, 0.86, and 0.79, respectively.

### MRI parameters

**Fig. 2:**
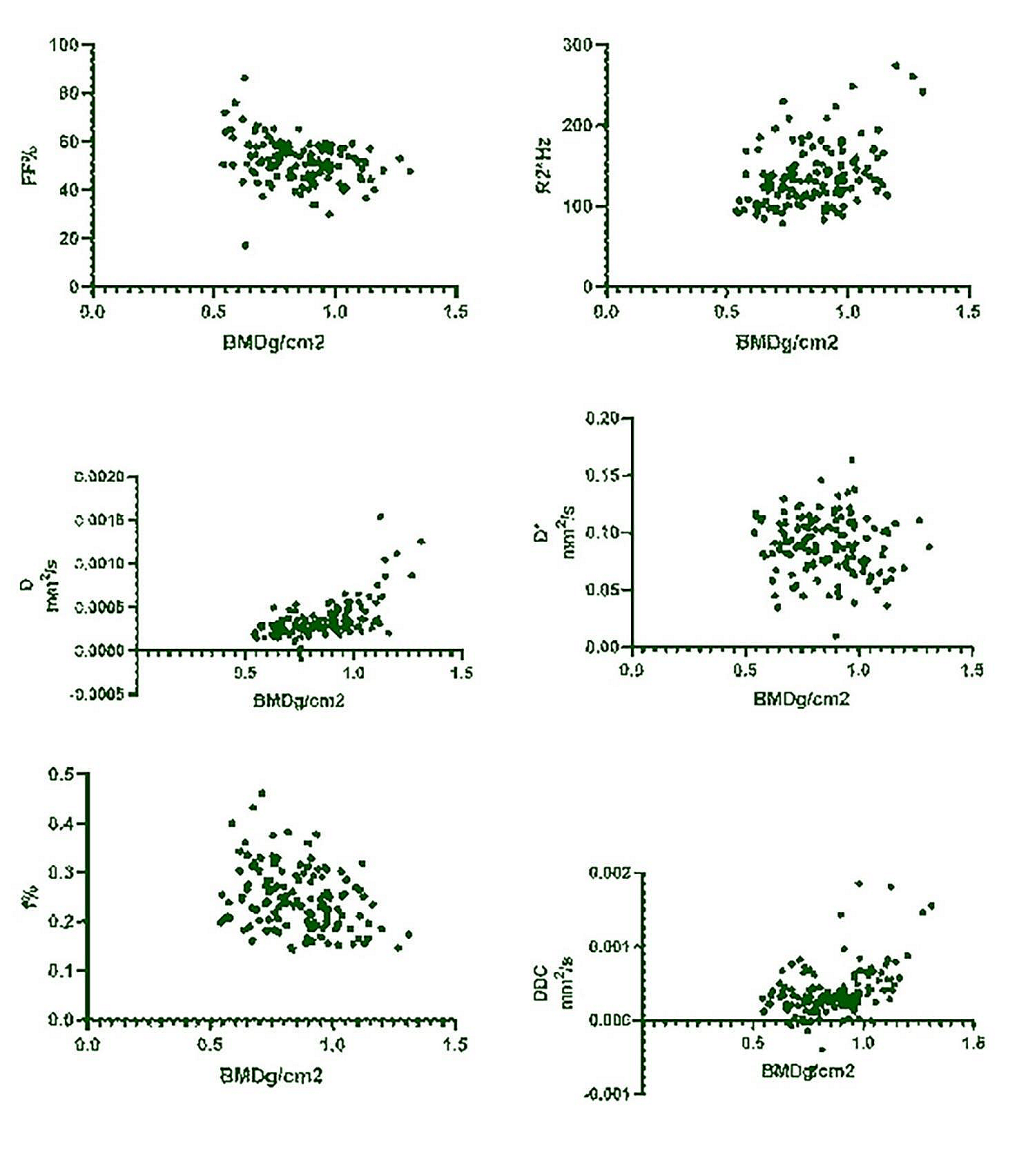
Scatter plots of parameters of each group

MRI parameters are summarized in Table 2. There were significant statistical differences in FF, R2*, f, D, DDC among the three groups. Afterwards, pairwise comparisons in these three groups were made. These results were also shown in Table 2. The box plots of comparison of parameters among different groups were shown in Fig. 2.

**Table 2:** Comparison of MRI parameters and BMI among the normal group, osteopenia group and osteoporosis group

Group	FF(%)	R2*(Hz)	D(10-^3^mm2/s)	D*(10-^3^mm2/s)	f(%)	DDC(10-^3^mm2/s)	BMD(g/cm2)	BMI(kg/cm^2^)
N group (*n* = 38)	49.83(10.43)	146.62(40.58)	0.48(0.31)	81.23 ± 23.84	0.22 ± 0.04	0.56(0.38)	1.04(0.13)	25.72 ± 2.45
Oa group (n=45)	50.87(9.76)	135.48 ± 30.71	0.32(0.15)	93.99 ± 29.79	0.24 ± 0.05	0.29(0.12)	0.89 ± 0.07	25.45 ± 2.64
OP group (n=52)	54.71(9.77)	120.55(38.55)	0.24 ± 0.09	90.30 ± 21.52	0.28 ± 0.07	0.25(0.26)	0.71 ± 0.09	23.87 ± 4.11
F/H	17.48	14.75	48.65	2.74	15.84	30.90	154.58	9.45
P	< 0.001	< 0.001	< 0.001	0.068	< 0.001	< 0.001	< 0.001	0.009
N and Oa groups(t or z, P)	-0.53 0.596	-2.14 0.032	-3.59 < 0.001	-	-1.51 0.132	-5.02 < 0.001	7.74 < 0.001	-0.50 0.615
N and OP groups(t or z, P)	-3.69 < 0.001	-3.75 < 0.001	-6.21 < 0.001	-	-3.86 < 0.001	-4.73 < 0.001	17.42 < 0.001	-2.74 0.006
Oa and OP groups(t or z, P)	-3.37 < 0.001	-1.87 0.061	-4.68 < 0.001	-	-2.50 0.013	-1.05 0.293	9.88 < 0.001	-2.44 0.015

Abbreviations: N in the table represents normal, Oa represents osteopenia, and OP represents osteoporosis

The D values and BMD values of the male group were higher than the female group, and there were significant statistical differences in D values and BMD values between the two groups, as shown in Table 3. The difference in BMD values between the group under 59 years old and higher age group was statistically significant, while the difference in other values was not statistically significant.

**Table 3:** Comparison of parameters of different genders

	FF value(%)	R2* value(Hz)	D value(10^− 3^mm^2^/s)	D* value(10^− 3^mm^2^/s)	f value(%)	DDC value(10^− 3^mm^2^/s)	BMD value(g/cm^2^)
Male (*n* = 54)	50.90(9.62)	136.31(40.27)	0.33(0.23)	91.24 ± 26.40	0.24 ± 0.052	0.33(0.37)	0.91 ± 0.17
Female (*n* = 81)	52.05(10.90)	138.96 ± 30.09	0.31(0.18)	85.00 ± 24.42	0.24 ± 0.06	0.32(0.25)	0.88 ± 0.14
T/Z	-1.608	-0.997	-2.304	0.916	-1.619	-0.975	2.808
P	0.108	0.319	0.021	0.361	0.105	0.330	0.006

Abbreviations: AUC in the table represents the area under the ROC curve

### ROC curves

**Fig. 3:**
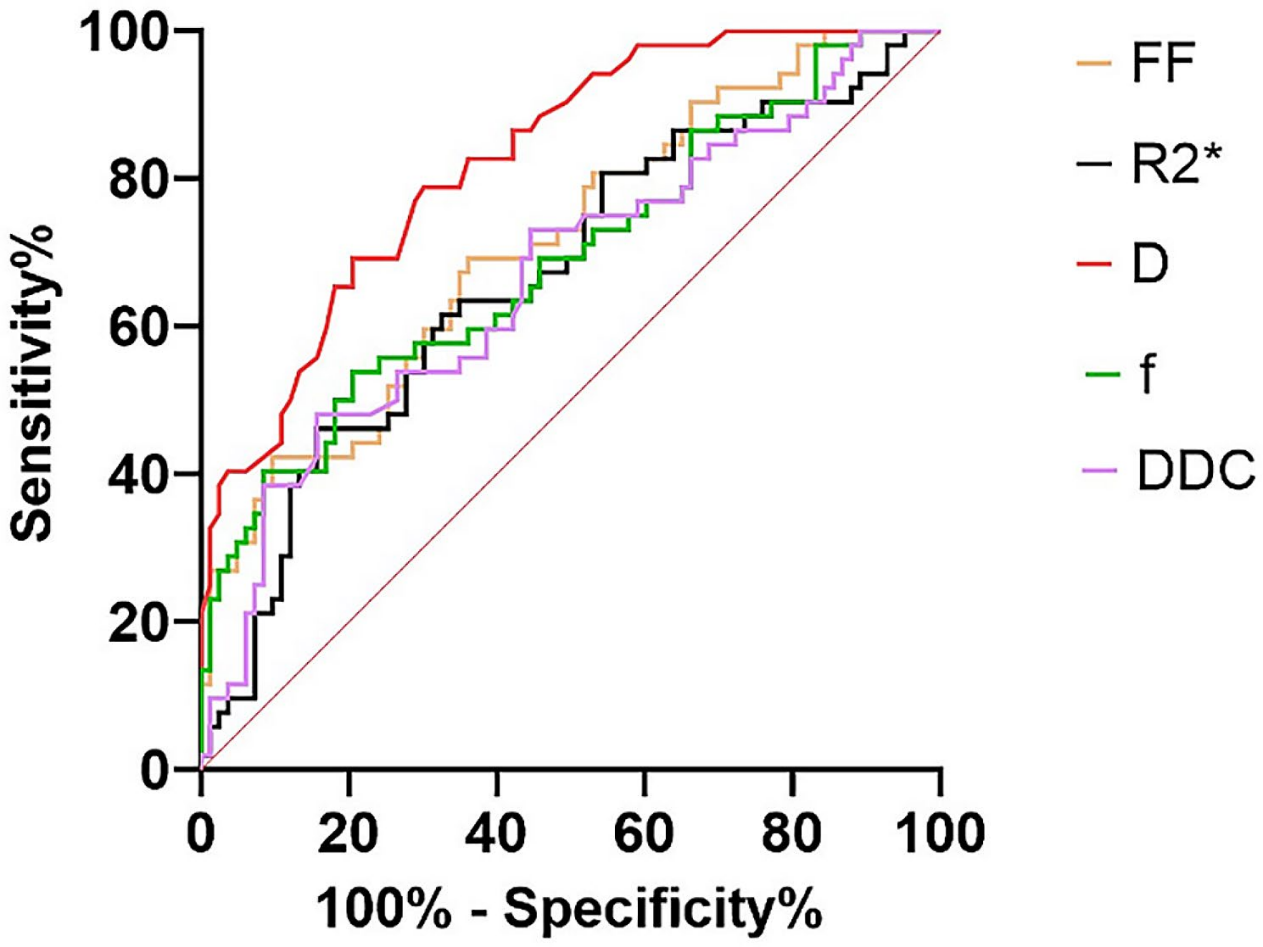
Comparison of ROC curves for various parameters

**Fig. 4:**
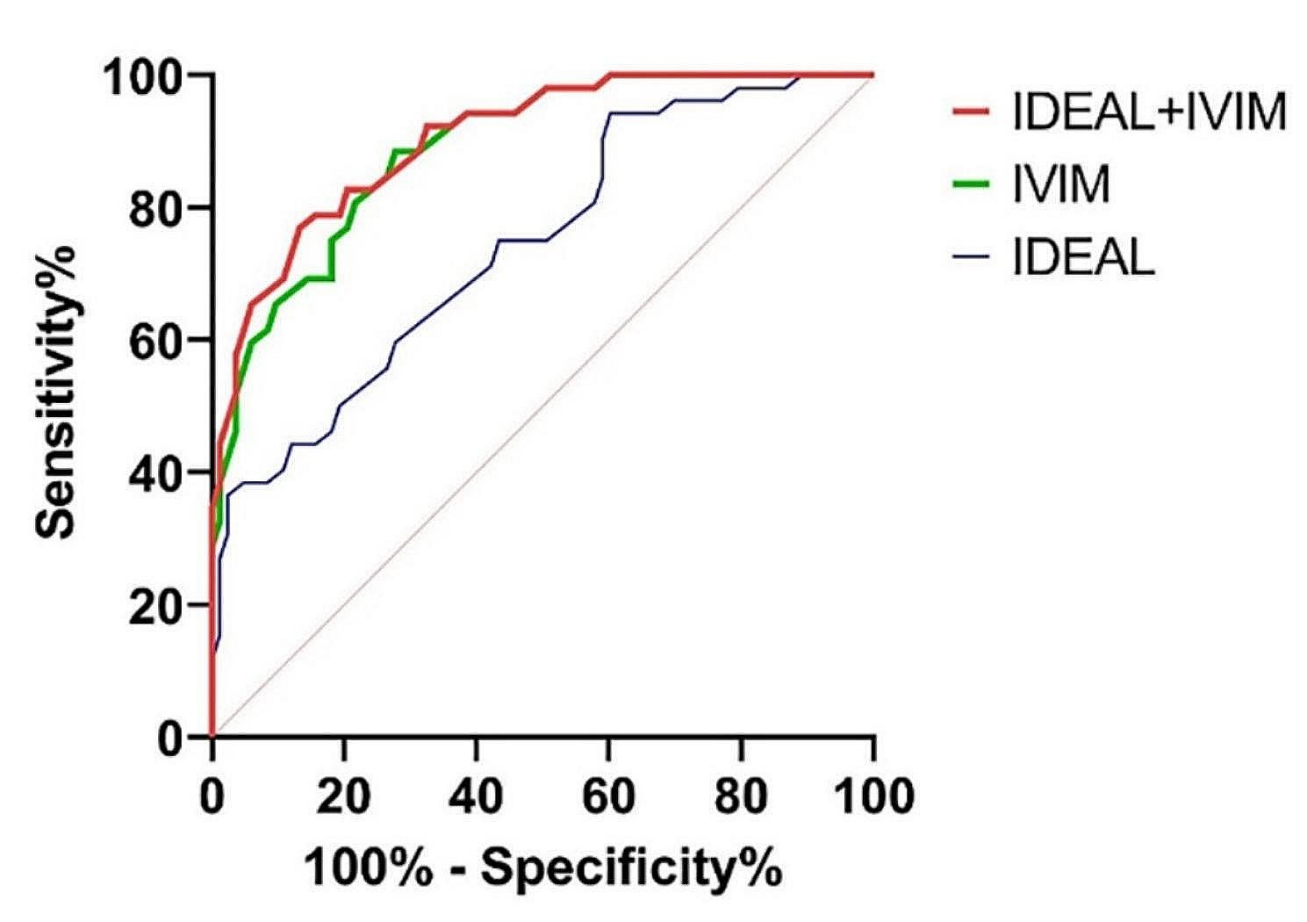
Comparison of ROC curves for the two techniques

After comparing the osteoporosis group and the other groups, the parameter values of the ROC curve were shown in Table 4; Fig. 3. The differences of AUCs were all statistically significant between D and other values (FF, *P* = 0.037; R2*, *P* = 0.004; D*, *P* < 0.001; f, *P* = 0.017; DDC, *P* = 0.006). We calculated the AUCs of IDEAL-IQ and IVIM-DWI, respectively, by combining their own values. Finally, we got the AUCs of combing the two sequences. These results were shown in Table 4. The differences of AUCs were statistically significant between IVIM-DWI and IDEAL-IQ (*P* = 0.001), combing the two sequences and IDEAL-IQ (*P* < 0.001) (Fig. 4), without significant difference between combing the two sequences and IVIM-DWI (*p* = 0.193).

**Table 4:** Comparison of ROC curve parameters

Parameters	Optimal value	AUC (95% CI)	Sensitivity (%)	Specificity (%)
FF value	51.93%	0.71 (0.62–0.80)	69.2	63.9
R2* value	113.81 Hz	0.67(0.57–0.76)	84.3	46.2
D value	0.30 × 10^− 3^mm2/s	0.83 (0.76–0.89)	72.3	76.9
f value	0.27%	0.69(0.60–0.78)	53.8	79.5
DDC value	0.23 × 10^− 3^mm2/s	0.67(0.57–0.76)	84.3	48.1
IDEAL-IQ	0.60	0.74(0.66–0.81)	36.5	97.6
IVIM-DWI	0.34	0.89(0.83–0.94)	88.5	72.3
IDEAL-IQ + IVIM-DWI	0.50	0.90(0.84–0.95)	76.9	88.0

### Correlation analysis

**Fig. 5:**
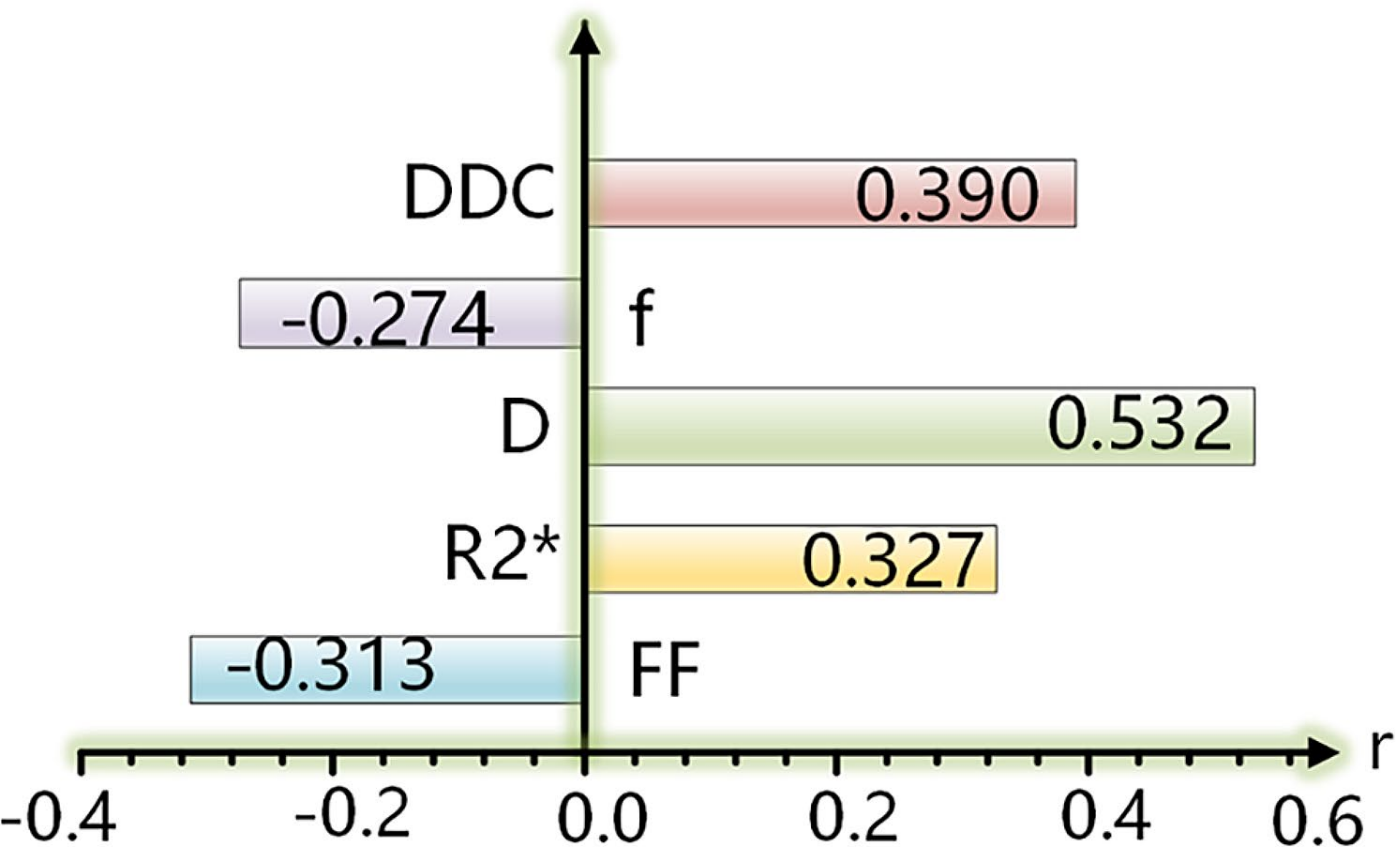
Comparison of correlation coefficients (r) of different parameters Abbreviations: The numbers in the crossbands were r values

FF value (*p* < 0.001), R2 * (*p* < 0.001), D value (*p* < 0.001), f value (*p* < 0.001), DDC value (*p* < 0.001) and BMD value were significantly correlated. The correlation coefficients (r) values were compared in the Fig. 5.

## Discussion

In our study, we investigated the utility of IDEAL-IQ and IVIM-DWI in evaluating OP. Our findings indicate that D and FF exhibited strong diagnostic performance for OP, with larger AUC compared to other parameters. With the exception of D*, all parameters demonstrated significant correlations with BMD. Moreover, when comparing the diagnostic efficacy of IDEAL-IQ and IVIM-DWI, we found that IVIM-DWI outperformed Ideal-IQ. This superiority may be attributed in part to the exceptional performance of the D value, which showed the best performance among all parameters. Additionally, several other perfusion-related parameters demonstrated their value for OP, thereby further enhancing the diagnostic capability of IVIM-DWI.

The FF serves as a reflection of the fat content within the bone marrow, with prior research suggesting a correlation between increased fat and decreased BMD [32]. However, our study found no statistically significant difference in FF values between the normal and osteopenia group, contrary to previous findings [33]. This inconsistency might stem from the incorporation of typical cases involving elderly individuals, where BMD naturally declines, leading to less pronounced observed variances. Nevertheless, our study revealed a negative correlation between FF value and BMD, aligning with findings from other investigations [33]. It may indicate that the increase of fat can reduce bone strength.

In our study, the R2 * value of the lumbar vertebral body exhibited a positive correlation with BMD. We hypothesize that as osteoporosis progresses, the magnetic field heterogeneity at the junction between bone trabeculae and bone marrow weakens relatively, particularly with the expansion of bone trabecular space, leading to an elongation of T2 relaxation value. Despite this, we found no statistically significant difference in R2 * values between the osteopenia and osteoporosis groups. This lack of distinction may stem from the inherent variability in spatial structures of bone trabeculae across cases, exerting an influence on R2 * value.

Our study revealed a progressive trend in the D value across the normal, osteopenia, and osteoporosis groups, with the normal group exhibiting the highest D value, followed by the osteopenia group and then the osteoporosis group. This observation was accompanied by a positive correlation between D and BMD values, consistent with prior research findings. The decrease in D value observed in our study may be attributed to an increase in fat content within the bone marrow, which fills and expands the space between bone trabeculae, thereby restricting the diffusion of extracellular water molecules. Additionally, this compression of true capillaries due to reduced space could result in diminished volume and inadequate perfusion of small blood vessels, exacerbating vertebral osteoporosis. Furthermore, we noted a decreasing trend in D value with advancing age, aligning with the progression of osteoporosis. While a previous study [34] reported no statistical difference in D values between men and women, our findings revealed a significantly higher D value in the male group compared to the female group. This difference could be attributed to the older age of participants in our study, leading to a pronounced decrease in BMD among female post-menopause. Overall, compared to other parameters, D may exhibit higher sensitivity in distinguishing between men and women and better reflect perfusion disparities between the sexes.

In contrast to earlier findings [35], our study did not reveal statistically significant differences in D*, which represents the diffusion effect, among the three groups examined. This discrepancy may be attributed to the limitations of small sample sizes in previous literature, potentially introducing statistical bias, compounded by the inherent instability of D* values. We hypothesized that the observed lack of differentiation in D* values among the groups could be due to the microvascular nature of the blood vessels supplying the lumbar spine. These vessels typically exhibit significantly lower blood flow velocities compared to larger vessels, resulting in a decrease in D* values and thereby minimizing differences among the groups relative to other parameters. OP has largely overlooked the DDC, which reflects the average diffusion rate within voxels. Prior studies have highlighted a strong correlation between DDC and the apparent diffusion coefficient (ADC) [25], an established parameter that, however, fails to distinguish between genuine water molecule diffusion and microcirculation perfusion effects. In our cohort, akin to ADC findings, we observed a positive correlation between DDC and Bone Mineral Density (BMD), suggesting that increased DDC may correlate with heightened tissue cell density within the examined region.

The f value, influenced by the microcirculation blood volume within tissue, reflects a significant parameter. However, our study diverges from previous findings [35, 36], revealing a negative correlation between f and BMD. This inconsistency may stem from a decline in vertebral microvascular perfusion levels, which chiefly affect f values. Nonetheless, BMD appears less affected by this factor, potentially resulting in a relatively low correlation between f and BMD. Interestingly, we found no statistical difference in f values between the osteopenia and normal groups, possibly due to the inclusion of older subjects in the normal group, exhibiting lower blood volume and flow velocities compared to younger individuals. Moreover, the lower T2 signal of lumbar vertebrae in the normal group compared to the osteopenia group further contributed to the decreased f value of the normal BMD group, consequently minimizing inter-group differences.

The limitations of this study include the exclusion of cases where DXA diagnosis is challenging and a limited sample size, thus restricting the generalizability of the results. Additionally, without the joint application of QCT (Quantitative Computed Tomography) for analysis, it is impossible to determine the correlation and statistical differences between various parameters and QCT results, and without further attempts to select the optimal b value, it may affect the accuracy of the parameters and the quality of the image. In conclusion, the integration of Ideal-IQ and IVIM-DWI sequences offers a quantitative approach to assess osteoporosis, aiding clinicians in their decision-making process. Among the parameters examined, the D value emerges as the most diagnostically effective in discerning osteoporosis, showcasing superior diagnostic efficacy compared to other values. Notably, the D* value demonstrates notably poor diagnostic performance, indicating its limited reproducibility for osteoporosis diagnosis. Age-related factors may diminish statistical disparities in certain parameters, while gender appears to significantly influence only the D value. Overall, IVIM-DWI may potentially outperform Ideal-IQ in terms of diagnostic efficacy for osteoporosis.

## Data Availability

The datasets used and/or analysed during the current study are available from the corresponding author on reasonable request.

## Abbreviations

IDEAL-IQ: Echo asymmetry and least-squares estimation-iron
IVIM-DWI: Intravoxel incoherent motion diffusion weighted imaging
DXA: Dual-energy x-ray absorptiometry
ICC: Intraclass correlation coefficient
FF: Fat fraction
D: True diffusion coefficient
D*: Perfusion-related diffusion coefficient
f: Perfusion fraction
DDC: Distributed diffusion coefficient
OP: Osteoporosis
BMD: Bone mineral density
DWI: Diffusion-weighted imaging
ADC: Apparent diffusion coefficient
