# Correction: IDEAL-IQ combined with intravoxel incoherent motion diffusion-weighted imaging for quantitative diagnosis of osteoporosis

**DOI:** 10.1186/s12880-024-01358-6

**Published:** 2024-07-24

**Authors:** Zhe Yang, Chenglong Liu, Zhaojuan Shi, Jian Qin

**Affiliations:** Department of Radiology, The Second Affiliated Hospital of Shandong First Medical University, Tai’an, Shandong 271000 China

**Correction to: Yang et al. BMC Medical Imaging (2024) 24:155.**

10.1186/s12880-024-01326-0.

Following the publication of the Original Article, the author reported that Table 2 of the Original Article contained an error. Some data in last two columns: BMD (g/cm2) and BMI (kg/cm2) are mistakenly switched.

**Incorrect**

**Table:** 

BMD(g/cm2)	BMI(kg/m^2^)
1.04(0.13)	25.72 ± 2.45
0.89 ± 0.07	25.45 ± 2.64
0.71 ± 0.09	23.87 ± 4.11
154.58	9.45
< 0.001	0.009
-0.50 0.615	7.74 < 0.001
2.74 0.006	17.42 < 0.001
-2.44 0.015	9.88 < 0.001

**Correct**

**Table:** 

BMD(g/cm2)	BMI(kg/m^2^)
1.04(0.13)	25.72 ± 2.45
0.89 ± 0.07	25.45 ± 2.64
0.71 ± 0.09	23.87 ± 4.11
154.58	9.45
< 0.001	0.009
7.74 < 0.001	-0.50 0.615
17.42 < 0.001	-2.74 0.006
9.88 < 0.001	-2.44 0.015

The original article has been corrected.

